# Causal inferences about others’ behavior among the Wampar, Papua New Guinea – and why they are hard to elicit

**DOI:** 10.3389/fpsyg.2015.00128

**Published:** 2015-03-10

**Authors:** Bettina Beer, Andrea Bender

**Affiliations:** ^1^Department of Social and Cultural Anthropology, University of LucerneLucerne, Switzerland; ^2^Department of Psychosocial Science, University of BergenBergen, Norway

**Keywords:** causal explanations, sociality, social cognition, kinship, Papua New Guinea, methodology

## Abstract

As social beings, people need to be able to interact intelligently with others in their social environment. Accordingly, people spend much time conversing with one another in order to understand the broad and fine aspects of the relations that link them. They are especially interested in the interactive behaviors that constitute social relations, such as mutual aid, gift giving and exchange, sharing, informal socializing, or deception. The evaluations of these behaviors are embedded in social relationships and charged with values and emotions. We developed tasks to probe how people in an unfamiliar socio-cultural setting understand and account for the behavior of others conditional upon their category membership – by trying to elicit the basic categories, stereotypes, and models that inform the causal perceptions, inferences and reasoning people use in understanding others’ interactive behaviors – and we tested these tasks among the Wampar in Papua New Guinea. The results show changes in the relevance of social categories among the Wampar but also, and perhaps more important, limitations in the translation and applicability of cognitive tasks.

## INTRODUCTION

As social beings, people need to be able to interact intelligently with those others who constitute their interactive environment (Sperber and Hirschfeld, 2004). Accordingly, people spend much time conversing with one another in order to understand the broad and fine aspects of the relations in which they and others engage. To understand others and to have better control about their own relations, they need enough information on the history and context of behaviors. Social interaction therefore depends on – and produces – a range of activities related to causal cognition: asking for explanations of behavior of other animate beings, construing possible causes and reasons, and/or ascribing responsibility for what emerges from this behavior.

Most people are especially interested in the interactive behaviors that constitute social relations: mutual aid, gift giving and exchange, sharing, informal socializing, deception, free-riding and so on. Social behaviors have moral characteristics that index and have consequences for particular relationships; people have definite expectations about who will or should behave in which way and these are often based on essentialist assumptions (Gelman and Hirschfeld, 1999; Gil-White, 2001; Sousa et al., 2002; Gelman, 2003; Waxman et al., 2007). Wampar, like others described in the ethnographic literature [see the special issue edited by Danziger and Rumsey (2013)], are sometimes circumspect about reading other people’s minds, but in many settings they are only too eager to discuss and evaluate the behavior, motivations and reasoning of others.

Our aim was to make explicit the information-searches and presumed causes concerning social behaviors by stimulating discussions with subjects using short scenarios intended to motivate people to reason about relations and motivations involved in the scenarios. We developed tasks to probe how people understand and account for the behavior of others conditional upon their social relations – by targeting basic categories and stereotypes (Hirschfeld, 1996), as well as the models and biases in causal attribution (Morris and Peng, 1994; Morris et al., 1995; Choi et al., 1999; Bender and Beller, 2011) and ascription of responsibility (Bender et al., 2007, 2012; Beller et al., 2009) that inform the causal perceptions, inferences and reasoning people use in understanding others’ interactive behaviors (Schlottmann et al., 2006). The tasks and results reported here were part of a pilot-study by the first author during her fieldwork among the Wampar in Morobe Province in Papua New Guinea (PNG) from March to May 2013^1^. The main goal of the study was to test if these tasks could be made relevant to local participants and hence could be used in a large-scale comparative study on causality and sociality. Our aim in this paper is to share the insights emerging from this process with regard to the difficulties encountered that may, but need not be specific to this field site.

In the following, we first provide some background information on the socio-cultural context of the Wampar, before describing the two studies that were conducted there, one employing an active information search with fictive scenarios on social behavior, the other using such scenarios to evoke evaluative responses. As it turned out that the main insight to be gleaned from these studies is not so much their empirical results, but rather the methodological problems they pose, the discussion focuses on those challenges that arise from this kind of cross-cultural research (cf. Baumard and Sperber, 2010).

## SOCIO-CULTURAL CONTEXT

**FIGURE 1:**
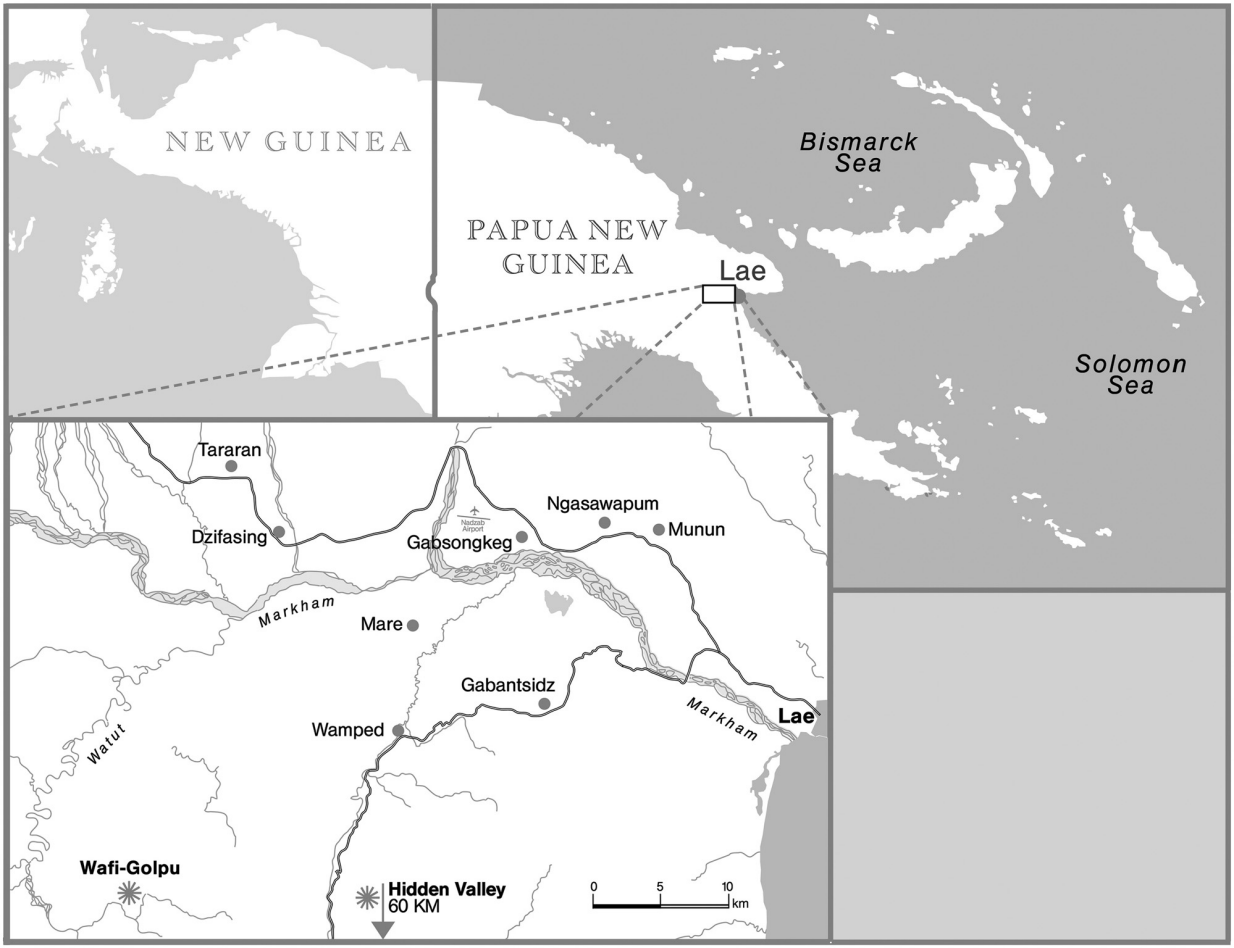
**Map of PNG and Wampar villages (map H. Schnoor)**.

The Wampar^2^ are a language group of about 12–15000 persons, occupying the area of the middle Markham River in Morobe Province of PNG (see **Figure 1**). They live in eight villages, five of them close to the Highlands Highway. The concentration of the population in villages is a post-contact phenomenon, developed under the influence of colonialism and Christianization after 1911. The Wampar practice of building houses in gardens away from the villages offsets this centralization in some areas, and in the last few decades many of these garden houses have developed into new hamlets away from the main village. With new economic opportunities through cash crops, cattle and chicken farms, and marketing along the main Highway, additional settlements have proliferated (Fischer, 1996, pp. 124–128). Today Wampar occupy an almost “suburbanized” area, with much of the population accustomed to engagement with the market economy.

Aside from the growth in number of hamlets and orientation toward the Highlands Highway (and market economy), there has also been an increasing factionalism in the dominant Evangelical Lutheran Church and the growth of new religious denominations and churches. Thus, the once centralizing force of a single institutional church as the center of village life from the early colonial period has been dissolved as well. Fischer (1975, 1996), who has studied the Wampar since the late 1950s, observed that until the 1970s, all Wampar conceptualized themselves as members of one of the about 30 named social groups called *sagaseg*. Wampar speak of *sagaseg* as patrilineal groups, but – as often happens in PNG – the incorporation of non-patrilineal kin is common. Also, the fusion of non-related *sagaseg* is historically verifiable. Furthermore, marriage patterns and practices have been diverse and are changing, with, for example, increases in interethnic marriages, children born out of wedlock and adoptions. Marriages within the same *sagaseg* were formerly subject to sanctions, but this is no longer the case, and some young people have even become unclear about their membership of a *sagaseg* (Fischer, 1996, pp. 129–144; Beer, 2006).

These changes (Beer, 2006; Beer and Schroedter, 2015) and others (including the very real possibility that a large gold/copper mine will be opened) have tended to challenge the hegemony of descent identities; what defines a Wampar, who counts as a member of the *sagaseg*, and how inter-*sagaseg* relations are configured are less clear than they once were. Fieldwork between 2009 and 2013 made it clear that kin networks, which now often join ethnically different groups, have complexified Wampar ideas concerning boundaries and significant social identities. In practice, the specific circumstances of particular social actors and the kind of relationships that they have among themselves and with their extended families, including those of interethnic marriages, have become decisive in accounting for commitments between individuals and groups.

## OVERVIEW OF THE STUDY

The study consisted of two parts: the first adopted the “active information search” paradigm (Frey et al., 1996; Huber et al., 2011) and comprised two short scenarios developed to probe naïve inclinations in the reading of intentions and behaviors of others, in contrasting types of behavior (helping and deception); the second part consisted of a narrated (fictive) scenario to evoke evaluative responses to behaviors of others and assumptions about the nature of categories and relations of the people involved. The order of tasks was the same for all participants, with Part 2 following Part 1.

In contrast to the majority of cross-cultural studies, we did not take a task that had been refined for usage with “WEIRD” (Henrich et al., 2010) samples, but aimed at formulating scenarios and questions relevant to the lived experience of social interaction in the local population under study to avoid what Medin et al. (2010) call the “home-field disadvantage.” We constructed stories of the sort, familiar to any social group, and especially also of non-WEIRD societies. As examples we chose behaviors which form the basis for inter-subjectivity and sociality, such as cooperation, commensality, and the morality^3^ of relationships, which are grounded in structured forms of interactions and on capacities as intention attribution, strategizing, or planned deception.

All tasks were written in English and translated into PNG’s lingua franca *Tok Pisin* (which is more and more frequently used among Wampar, especially between Wampar parents and their children, and most of the time in interactions with non-Wampar), but were presented verbally.

## PART 1: ACTIVE INFORMATION SEARCH FOR SCENARIOS ON SOCIAL BEHAVIORS

The main goal of Part 1 was to investigate which type of information Wampar consider to be essential for venturing causal explanations of the course of social interactions. It therefore amounted to an active information search task, in which the presentation of a target question (on distinct social behaviors) was aimed at generating further questions (e.g., about the persons involved, their relations, or the situation) relevant to the evaluation of the behavior described in the scenarios. We also wanted to know what initial reason/causes people imputed to the characters described in the scenarios.

### METHODS

#### Participants

Twelve Wampar from the village of Gabsongkeg participated in this part of the study (five women, six men, and one schoolboy), but its analysis is confined to the adults. The trial interview with the 7-years old schoolboy generated only one answer, which was not to the point: he commented on his own past behavior^4^. The results are therefore reported for 11 participants (age *M* = 40.0 years, range: 18–73). All of them went at least to elementary school and were involved in farming and some small business. More information about biography, education, and family background of all participants is available because the ethnographer has known them since 1997. The interviews were relaxed and all participants were free to discuss personal and/or problematic topics.

#### Material

The task revolved around two target scenarios, each followed by a set of three questions. The scenarios focused on the social interaction of “helping” and “deceiving,” respectively:

(A) “X helps Y to finish some hard and boring work:”
(A1) “Why do you think X helped Y?”
(A2) “Ask me questions: what do you need to know to answer the question why he/she helps?”
(A3) “How would you say other people (living in your neighborhood/village) would explain why X helps Y?”
(B) “X deceives Y by not giving him his share of the proceeds of a joint business/work”
(B1) “Why do you think X deceived Y?”
(B2) “Ask me questions: what do you need to know to explain why X does that?”
(B3) “How would you say other people (living in your neighborhood/village) would explain why X does this?”

X and Y were replaced either by local male and female names or by “a man” or “a woman.” When necessary the interviewer gave for “hard and boring work” local examples like carrying something heavy, or cleaning a big garden.

The first questions (A1, B1) aimed at finding out how people reason about the described behaviors. The second questions (A2, B2) were connected to the first and are very open; they tried to identify what information people ask for if they feel uncertain about the reasons for the behavior. The third questions (A3, B3) aimed at getting access to participants’ ideas about shared (and non-shared) desires, beliefs, and reasons for behavior.

Questions 1 and 3 thus directly targeted causal explanations, the latter with a focus on sharedness. We expected that mutual aid was explained more often in terms of balanced and generalized reciprocity, specificities of the situation, and less often by individual characteristics of personality or in terms of market exchange. Question 2 was intended to produce data on the information people considered most relevant to establishing causal explanations. Here, we expected people to ask either for attributes of the category of people involved (such as sex, age, or ethnicity), their personal attributes, and information about the relation they have, or for more details about the situation.

#### Procedure and design

All participants were given both scenarios with three questions each in the above order; scenarios were read identical or very similar to the original text; eight of the 12 interviews were fully recorded. Furthermore, the ethnographer made detailed notes on the situation and context, and recorded other pertinent observations, in a field notebook.

### RESULTS

As indicated above, the prime concern of this part rested on question 2 and on the data it would procure regarding active information search; this is presented first. Findings from questions 1 and 3 on the explanations for the behaviors are presented afterward, separately for scenarios A and B.

#### Active information search

With respect to its main aim, the investigation of active information search, the questions about helping or not-sharing (A2 and B2) were a failure. When asked what one needed to answer the target question, literally *every* participant simply repeated the target question. When the ethnographer explained that they could ask for any further information, nobody requested any. These questions seemed to be unintelligible or too abstract. Participants made clear that they took it that the question itself sufficed to produce an answer, and, if it did not, other questions could not help. To ask in roundabout ways for further information so as to get to an answer (like in a quiz game), which one could get directly, did not make any sense to the participants.

#### Explanations for the behaviors

Talking about the scenarios gave some important insights, nevertheless; yet, they were different from what we expected.

*(A) Helping scenario.* The first question about the first scenario, in which person X helps person Y (A1), was answered by eleven people. One man was excluded from the analysis because he did not address the question. Answers of the other 10 participants can be grouped as follows (see **Table 1**; more than one answer possible).

**Table 1:** Explanations for social interaction: helping.

Response categories (with concrete responses)	Frequency	
	In numbers	In %
**Balanced reciprocity**
Y helped X in the past or is expected to help X in the future	5	
Y provided food for X	1	
X wants to marry Y’s daughter	1	
Subtotal	7	36.8
**Generalized reciprocity**
X is feeling sorry	3	
Y is alone	1	
Y is weak and tired	1	
Subtotal	5	26.3
**Market exchange**
Y gave money to X	3	
Subtotal	3	15.8
**Dispositions of X**
X has special skills/knowledge	1	
It is X’s manner [*pasin*] to help	1	
Subtotal	2	10.5
**General evaluation**
This is good or good behavior [*pasin*]	4	
Subtotal	4	21.1

*Total*	19	100.0

The most frequently given answer, that helping is based on balanced reciprocity, was expected as it is a common feature of sociality in PNG (cf. Tracer et al., 2014). Several respondents located the reason for X’s behavior in the situation based on a more generalized reciprocity in which intragroup exchange is organized by an ethic of as-needed assistance. The spontaneous first answer of three respondents, who assumed that Y had paid X to help him, was less expected, but might be indicative of an increasing integration of the Wampar population into market economy. Only two participants mentioned X’s disposition.

The question on what other Wampar may think about the situation (A3) was answered by the same 10 participants. One said he only knows what others think if he can talk to them. Another respondent (a much criticized businessman who leases Wampar land to non-Wampar migrants) inquired whether the question referred to what people think about his own business^5^. Five assumed that others would answer as they had and merely repeated what they had said – with only little variation, or with additional reasons for their response. Five respondents said that there are many different social behaviors and mindsets. Three of this latter group emphasized ongoing social transformations, largely caused by the introduction of a money-based economy; they complained that today only money counts and that people become more egoistic and lazy, and/or they only focused on their own nuclear family referring to the conflict between communal and individual values (Barker, 2007, pp. 9ff.).

*(B) Deception scenario.* Questions on this scenario were answered by 10 participants (see **Table 2** for an overview; more than one answer possible).

**Table 2:** Explanations for social interaction: deception.

Response categories (with concrete responses)	Frequency	
	In numbers	In %
**Balanced reciprocity**
Y deceived X in the past	1	
Subtotal	1	4.8
**Action of X (attributed to circumstances)**
Money has changed the way people think	1	
X is lying (for a specific reason)	2	
X needs the money for realizing a plan	4	
Subtotal	7	33.3
**Dispositions of X**
X is selfish/greedy	5	
X is lying (as a habit)	1	
X is lazy / does not like to talk	2	
Subtotal	8	38.1
**General evaluation**
This is bad or bad behavior [*pasin*]	4	
Subtotal	4	19.0
**Other**
Y should have tried to find out by himself	1	
Subtotal	1	4.8
*Total*	21	100.0

The reactions of participants to the first question (B1) were split like in the helping scenario: eight respondents located the reason for the behavior in the disposition of person X. One participant mentioned the transformative power of money as a cause of deception as it changes the way people think and their social behaviors. The answers of other participants, who stated what X is doing, can be interpreted in a similar direction. They emphasized the circumstances and his desire, which explains his behavior, rather than characterizing him as a person. This resonates with everyday experience during fieldwork: when somebody took food, tools or other things from somebody else, the ethnographer was often astonished that people got very angry about what happened, yet did not blame the person or accuse him or her of possessing negative character traits. For example, a young man once stole cooked food that an older woman had put aside to be eaten in the evening. This is thought of as extremely bad, disrespectful behavior, and the woman’s family got very angry. But, even when they found out who it was, the incident was explained in terms of circumstances (he had been drinking, and become hungry) rather than by character deficits in the young man. Mostly, deception, stealing, and violent behavior were quickly forgotten and had few consequences for the evaluation of the person in the future. One respondent even blamed Y because he should find out himself about the money and not rely on X giving it to him.

The question on how fellow Wampar would reply (B3) was answered by 10 participants. Those who did answer the question in the intended way were split: three replied that other Wampar would give the same answer and four replied that they would evaluate the situation in different ways. A woman made very clear (like some participants after the first scenario), that “lifestyle” has changed; she said: “Everybody follows his wife only and does not share anymore (*bihainim meri tasol*, means looking after their own family). Selfishness has become very common.”

### DISCUSSION

In general, the findings from the first part of the study were informative with regard to the sociocultural dimension of the task (i.e., the attitudes and expectations involved or activated), but less so in terms of information search: while we did obtain data on the content of causal explanations, obtaining data on the processes involved in causal reasoning was more difficult.

The causal explanations used in the helping scenario corresponded partly with what we had anticipated, based on our (anthropologically informed) picture of Wampar society and the ongoing changes in their life-style (see Socio-Cultural Context). Reciprocal relations are the links in the chains constituting the fundamental relations of social networks. The principles of balanced and generalized reciprocity are internalized early in life and these thematize many types of action. Interestingly many Wampar are very aware of the transformation of intentions and motivations that has accompanied their increased integration into market economy: nowadays some Wampar actively try to avoid or curtail the reciprocal obligations that had been central to their community^6^. Several participants emphasized the transformative power of money, which encouraged people to refuse help to others who could not pay, so that inequality also becomes more pronounced. If people do not have any money they must offer work or something else as ‘payment’ instead. Some Wampar complained that mutual help in the context of generalized reciprocity and community values has become rare (cf. Barker, 2007, pp. 8–12).

In many answers, money is itself assigned a causal role in social behaviors and their transformation. The desire for money and things is here a causal force, which is less located in the weakness of a person – following the notion of a ‘personality’ (Goldie, 2004) – but rather in outside powers and circumstances. Behavior in the deception scenario was explained along these lines: somebody with a plan to buy or do something, or a strong specific need for the money, is understandably motivated to deceive. This resonates with comparative studies of American and Chinese attributions of causes, which suggest differences between dispositional and situationalist reasoning about social events (Morris and Peng, 1994). It also evokes certain observations reported by Tracer et al. (2014, p. 191) of the ultimatum and the third-party punishment games as played by Au speakers in PNG: “Several player 2 s expressed concern for the plight of player 1: ‘It’s all right, maybe he really needs it and has some work he has to do with it,’ one said, and yet another asserted, ‘It’s not good, it’s not a good split, but I don’t care, he probably has a reason.”’

The change of social relations and the attendant diversification of values was another topic repeatedly raised by participants. Therefore making claims about social behaviors and the reasons behind them among “the Wampar” has become even more difficult than it might have been in former times. Reflection by many Wampar on specific changes of values and behavior facilitates discussions about shared (and non-shared) desires, beliefs and reasons for social interactions. Our scenarios and the related questions were starting points for discussion, although more detailed and committed discussions happened in informal situations and in small groups of people who know each other well.

Although it is clear when people’s exclamations express their own moral attitude with a very general evaluative response, *Em gutfela (pasin)!* (“This is good [behavior/manners]!”), central values might or might not be attributed by participants as a cause for behaviors. This lack of clarity is exemplified in the spontaneous answers to the question “Why does X help/deceive Y?” In the helping scenario, for example, these responses were often not directly connected to causal reasoning, in the sense of “X does it because it is good behavior.” In the deception scenario this is particularly clear; participants often responded with a similarly evaluative statement, “This is bad behavior,” without stating or implying anything about the *reasons* for the behavior.

The dominant strand of research on causal cognition is basically concerned with the processes of perception, learning, and reasoning about abstract causal relations (Michotte, 1963; Bender and Beller, 2011; Waldmann and Hagmayer, 2013). In social contexts, attributing causal involvement in an event is often intertwined with a moral dimension (Samland and Waldmann, 2014) and with the ascription of responsibility for that event (e.g., Heider, 1958; Shaver, 1985; Hewstone, 1989; Weiner, 1995). These concerns appear to be reflected in the explanations of the Wampar participants for the behavior of person X, some of which we tentatively categorized as ‘dispositional’ (i.e., all those that refer to the manner or personality of X in **Tables 1** and **2**), while other explanations we categorized as referring to circumstances that triggered them. Even less clear is the categorization of those cases that reflect balanced reciprocity: these explanations seem to presuppose both a situation of on-going exchange and a willingness of X to respond to this strongly normative relationship, as the joint causes of his current behavior. More importantly, however, the explanations seem to reflect a concern with the still important relational dependencies among the protagonists. Please also note that dispositional explanations are much more frequently given to account for negative behavior (deception) than positive behavior (helping).

Investigating the extent to which relational dependencies are shaped by information on social categories such as kinship was one prime goal of this task. In particular, we had assumed that participants would be interested in collecting information that they considered relevant for an account of the event, thereby revealing salient categories. However, to accurately evaluate a person, relation, or situation by systematically collecting information was not an aim of any of the participants – at least not in the way we expected. Rather they used examples from their own social environment to make sense of the scenario (cf. Stenning, 2012). Three participants were clearly motivated in their answers by their own personal situation and/or relation to the ethnographer. The active information search task was therefore not successful in revealing the exploratory processes that people use. It also raised the question of whether people are as interested as we assumed to uncover causes behind behavior in order to evaluate it. Are reasons or causes for behavior really necessary to understand, evaluate and respond to others with whom they are in relations? If people do not assume that somebody has a constant personality constituted by lasting characteristics, which have to be uncovered to anticipate future actions, the motivation to explain causal connections between personal attributes and behaviors might be lower. To explain behavior by circumstances opens up a wide spectrum of possibilities which participants did not discuss for fictive scenarios but connected to the specificities of well-known social situations.

## PART 2: SCENARIO EVOKING EVALUATIVE RESPONSES

The main goal of Part 2 was (a) to investigate further what defines and maintains relationships between people, especially kin (e.g., emotional closeness, physical substances, commensality, or sharing of food, growing up together, teaching and socialization, or procreation), and (b) to scrutinize what Wampar saw as causes of emotions and subsequent actions relevant to moral evaluations such as punishment. In order to evoke such evaluative responses, we crafted two fictive scenarios, one involving incest and one patricide, which are likely to be areas of strong moral feelings and evaluations. In the course of this study, however, it became clear that the (intense) discussion on the first scenario would take too much time to follow this up with a second round. This section is therefore confined to the incest scenario.

### METHODS

The same participants were interviewed as in the first study, except for the schoolboy and a man of 35 years, with whom the interview was interrupted (thus rendering a total of *n* = 10 participants; age *M* = 40.5 years, range: 18–73).

#### Material

The task focused on one target scenario revolving around incest prohibition in several versions with changing types of kin, each followed (ideally) by a set of 10 questions. The basic scenario described a situation in which close relatives of opposite sex feel attracted, have intercourse and have a child together. The first version featured a mother and her son:

“A young man was stolen as a baby and taken to a distant town, where a family adopted him. He grew up as a son of the family. He never learned anything about the family into which he had been born. One day, when he was grown up, he came to his birth village. Here, he happened to meet his still young mother, who was a widow. The two fell in love, she got pregnant and they had a child. People found out that they were related. There were many heated discussions about what had happened and everybody started talking about it. What do you think people said?”

The second version exchanged sister for mother and was not read out in its entirety, but was just repeated with the main information staying the same.^7^ The narratives were followed by a series of questions that can be clustered into three groups:

(I) Moral evaluations and their sharedness
(1) What do you think the people of the village are saying? (And what might his/her relatives think/say?)
(2) How do the man and his mother [sister] respond when they are confronted with what other people say?
(3) How would you say the man and the woman feel about what happened?
(4) Is the son [mother/brother/sister, respectively] a good or bad person? Are they equally so? If so why?
(II) Essentialist notions of persons and their relations
(5) The story states that they did not know they were related; do you believe that? Would it be possible to recognize relatives you have never met before? Would they have intuitively felt that they are related?
(6) Do you think it is possible that the young man became more similar to his adoptive family than to the one into which he was born?
(7) What characteristics do you expect the baby to have?
(III) Practical consequences of moral evaluations
(8) Do you think the baby should be adopted by somebody who lives a long way from the village?
(9) Will this child become a bad/good or unsuccessful/successful man as an adult? Why?
(10) Do you think the young man should stay in the village or leave?

In each section, participants sometimes gave no answer or answered earlier questions, when confronted with a new question and vice versa; this is discussed below.

#### Procedure and design

It was planned to read all scenarios (and variations of them) in the same order to each participant, each followed by the same series of questions. It turned out, however, that only the first version of the (first) scenario could be read in its original version. A second reading with variations (i.e., with different kin relationships between the partners) or about a new topic (i.e., the originally planned second scenario) would have been too long and boring for the participants^.^ For example, participants grew impatient when asked to listen to the same initial sentences again as the ethnographer tried to test the variations in kin relations. Accordingly, she changed the procedure for the second round and only asked informally how the participant would react if the protagonists were related differently.

The first scenario was read to all participants, and the first question was always the same for all participants. The following questions had to be modified, simplified, and adjusted to the conversation for reasons discussed below. However, the gist of the questions remained the same; they were only less differentiated and repetitive. For example, it did not make sense to differentiate between the general gossip and what close relatives said, so that the second part of the first question was left out. Sometimes participants thought either the interviewer did not listen attentively enough or did not understand their answer if she asked “the same” – in fact slightly modified – question again, which irritated and annoyed some participants and made them impatient.

### RESULTS

Participants’ responses are presented in the same order of the three groups.

#### Evaluations and their sharedness for the mother/son-version

The first question (Qu.1, see **Table 3**) what other people would say about the events described in the scenario was answered by four participants immediately by affirming that there would be *a lot of gossip* but without being precise about the content. Four emphasized that other Wampar would get angry because it is his real mother, two participants blamed the woman (or said other Wampar would blame her), that she should have found out more about the man before having sexual relations with him. While these types of responses mainly expressed a negative evaluation, four were concerned with practical implications instead; three of these assumed people would say the couple should marry, one they should separate although a separation would raise the question of who looks after the woman and her child^8^. A woman described different opinions, including indifference about social behavior of others, which she blamed on social change and the loss of the values associated with generalized reciprocity. The answers show that attitudes are diverse and changing among Wampar: participants consider a wide range of conditions for the described behaviors and are reflexive about the diversity of possible moral evaluations. Because the narrative provoked immediate evaluative responses many seemed to find it difficult to change perspective to report what they thought others would have said.

**Table 3:** Responses on moral evaluations and their sharedness (cluster I).

Response categories (with concrete responses)	Frequency	
	In numbers	In %
**(Qu.1) Moral evaluation attributed to the people of the village**
Assessment as bad behavior	14	56.0
Concern with practical implications	4	16.0
People’s opinions will be diverse, some indifferent	3	12.0
It doesn’t matter anymore	2	8.0
Focus on positive aspects	2

*Total*	25	100.0
**(Qu.3) Feelings attributed to the couple**
They were ashamed/felt bad	9	69.2
They will stay together	3	23.1
They did not worry	1	7.7

*Total*	13	100.0
**(Qu.4) Participants’ own evaluation**
Positive (because they did not know)	4	50.0
Negative	3	37.5
It doesn’t matter anymore	1	12.5

*Total*	8	100.0

One example shows that the interpretation of answers needs to be understood in terms of the particulars of the everyday life. A woman first said that everybody in the village would get angry, and then exclaimed: “It must be LOVE! They should marry.” She used the English word ‘love,’ unlike any other participant. She answered the second question (what the couple thinks about the gossip), and added, “They won’t worry about gossip and won’t follow what other people say.” When asked about her own evaluation of their behavior, she replied: “They are happy because they do not listen what others say. He must have come back to the village with lots of money.” Her statements painted an unusual picture of an intense love story. It turned out that she interpreted our scenario in terms of her favorite Nigerian (“Nollywood”) soap opera *True Love*.

The question how the couple felt about what other people said (Qu.3) was answered (except in the above described case) by most participants consistently: that they felt ashamed, “bad” or “sorry.” With respect to their own evaluation (Qu.4), participants were split (three replied that mother and son are bad people, because what they did was wrong; four said that they are good people, they did not know, what they were doing).

#### Essentialist notions of persons and their relations for the mother/son-version

We also wanted to probe how participants conceptualize the relatedness between mother and child and asked if the two could have known that they were related (Qu.5, see **Table 4**). Four participants answered with a clear “no.” Two replied that being kin was the cause of their attraction (meaning they noticed something), but that mother and son confused affinity between kin and sexual attraction. Two respondents explained that the mother should have felt it because of her love for the child.

**Table 4:** Responses on essentialist notions of persons and their relations (cluster II).

Response categories (with concrete responses)	Frequency in numbers
**(Qu.5) Possibility to know relatedness**
Yes	4
No	4
Don’t know/cannot know	1

*Total*	9
**(Qu.6) Similarity with foster family?**
Yes (even if only behaviorally)	3
Not sure/no answer to the question	7

*Total*	10
**(Qu.7) Characteristics of the baby**
He will be good	6
He will be bad	1
Depends on the strength of parents’ belief	1
Don’t know/cannot know	2

*Total*	10

To find out how belonging creates similarity or difference, and changes the quality of relations, we asked if the boy might have become like his foster family (Qu.6). Two respondents answered “yes” but did not clarify in which ways, one said his *kastom* (culture, tradition), *pasin* (behavior, manners) and relations have become the same as his foster family’s but that he still looks different from them.

The next question focused on the child of the incestuous relationship and what characteristics it might have (Qu.7). Answers to this question showed the highest agreement between participants^9^. Most said that it will be a “good child,” but qualified their response in different ways. Only one answered that because of the blood – according to *kastom* – the child would be “bad.”

#### Practical consequences of moral evaluations for the mother/son-version

In respect to how the baby should grow up (Qu.8, see **Table 5**) opinions diverged. Seven of the participants said it should stay with the parents or the mother, while three thought it would be better to send it away, at least until it became an adult. On the other hand, only one of the participants thought the couple should remain together (Q.10).

**Table 5:** **Responses on practical consequences of moral evaluations (cluster III)**.

Response categories (with concrete responses)	Frequency in numbers
***(Qu.8) Where should the child live?***
With the parents	5
With the mother	2
Should be adopted/move away	3

*Total*	10
***(Qu.10) What should the couple do?***
Move away to the town where he grew up	3
Separate	3
Follow their own feelings	1
Stay together in the village	1
It doesn’t matter anymore	1

*Total*	9

The variation featuring a brother and his sister as incestuous partners (X2) provoked interesting responses, with an increased number of participants ready to emphasize their relatedness by blood. Two participants rated the case as bad as that in the first story, while three said it is much worse than incest between mother and son. All five reasoned that the love between mother and child is stronger in the mother/son-version and that their blood is, in the case of real siblings (opposed to cousins or parents and their children), even more similar or identical.

### DISCUSSION

#### Moral evaluations, their sharedness and practical consequences

The general evaluation of the events described in the scenario (assessed with the first cluster of questions) was unanimously negative. With regard to the involved persons, however, the moral evaluations diverged. Half blamed the couple (some more specifically the woman), while the other half said that the couple was not responsible because they did not know the truth. Notably, many participants shifted focus from why this happened to practical solutions for the outcome, and some refused to make any attributions whatsoever. In terms of attribution theory, the first two types of responses reflect distinct tendencies: one the tendency to personally blame the actors involved, and the other the tendency to consider mitigating circumstances such as lack of knowledge.

The third type of responses appears to be linked to a widely reported disavowal of interest in reasons and responsibility for action in the societies of the Pacific and other parts of the world (for an overview, see Träuble et al., 2013). In addition, an ‘opacity of mind’ has been described specifically for parts of Melanesia (see Rumsey and Robbins, 2008 and the papers therein), that combines (or substitutes) the disinterest with a reluctance to attribute mental states and motives to others, based not (only) on disinterest, but on respect for others’ privacy and autonomy, or on fears how knowledge is used (for a discussion of the literature, see Laidlaw, 2013, pp. 158–159).

This raises an important question: are reasons and causes for behavior really *necessary* to plotting the personal and political consequences of those behaviors? Most of the participants took a very pragmatic line of arguing in that they seemed to be not very interested in the question *why* something happened (the attraction, the reasons for the confusion etc.), but more interested in and worried about the outcomes. How should the community deal with deviant behavior? And how should relatives handle the results and outcomes? The focus on questions like these has led to the characterization of Melanesians as ‘pragmatic’ (cf. Barker, 2007), but this pragmatism does not need to displace considerations of morality. One has to understand the socio-cultural context of pragmatism, as Read (1955) described in one of the first studies of morality in PNG, specifying that their pragmatism is given shape in specific cultural conceptions of the person and thus may vary according to the relationship in play (Barker, 2007, p. 6).

As in Part 1, the results of Part 2 indicate changes in the morality of kinship among the Wampar, but they also underline the difficulty of controlling the social setting well-enough to investigate cognitive responses formally (reproducibly) and effectively. Most respondents were influenced strongly by known cases in their social world. In addition to the case of “Bubu-Dadi” and his offspring, other instances of controversial marriages (for instance, between classificatory siblings^10^) influenced people’s evaluations; such cases also led participants to abandon rumination upon fictional moral questions in favor of discussion of actual people and their behavior.

#### Essentialist notions of persons and their relations

The question of kin relations was answered in many different ways. Wampar ideas about the transmission of physical, mental, and moral qualities from parents to children are vague. Many still explain that blood determines children’s affiliation to the lineage and clan of their father (as one participant in our sample explicitly did). However, most of them add that many exceptions exist and that nobody is really sure how corporeal inheritance works. Some ideas may still be based on Wampar conception theories that Fischer (1975, p. 128) described for the 1960s: the man gives (*erem*) his wife the child and she carries (*epeng*) it. The relationship between father and child is hence more important for descent than that between mother and child. The child receives his or her own blood (*wi*) only from the father, but an emotional and bodily bond (the child is formed in the uterus) between mother and child – developed through the uterus (*wawang*) during pregnancy – is also thought of as important. This theory is used in pragmatic ways, with little regard for coherence of doctrine, particularly when it comes to interpreting the belonging of children of interethnic marriages (Beer, 2006; Bacalzo, 2012). In the answers to our questions on the nature of social bonds, Wampar were less essentialist than we had assumed and more pragmatic or situationalist (“they did not know, so let them be happy together”). The bonds between social/biological mother and child were emphasized in many answers to our questions about where it should grow up, or about the influence of foster parents, and some explained that the attraction between mother and son emerged because mother love and sexual attraction had been confused.

Most participants reasoned that an incestuous relationship between siblings would be much worse than one between mother and son who, on Wampar views, do not share blood even though the relationship remains forbidden, because she has carried and given birth to him. Some tendencies in the evaluations of behavior and reasoning processes behind it are worth mentioning. Aspects of family values and gender relations have been articulated in several statements: if one of the partners is to be blamed, it is the woman and not her son. She should have inquired about his background before beginning a relationship, and it was assumed that she would be more likely to feel that this is her child, because of a special bond between mother and child. This also resonates with pragmatic problems Wampar emphasized: who would look after her and the child? And how are the child and his parents placed in the kinship system? The degree of sharedness of evaluations of relations and sociality among Wampar is another important aspect. Even from a small number of interviews the dimensions of sociocultural change and its consequences have become obvious in the diversity of answers from participants and their reflections on this period of social transformation. Wampar seem to support Barker’s (2007, p. 12) generalization: “Coastal areas that have had the earliest exposure to colonial rule and are most deeply integrated into national and international networks tend to be more tolerant of moral ambiguity.”

## METHODOLOGICAL PROBLEMS

It is widely agreed that, ideally, adequate psychological/cognitive testing requires cross-cultural research (Bender et al., 2010; Henrich et al., 2010; Medin et al., 2010; Beller et al., 2012). The need to combine the controlled experiments commonly used in psychology and the interpretive ethnographic research central to anthropology has also been underlined (Beller et al., 2012). Yet doing so is not easy, especially in the absence of details concerning the practical problems, theoretical traps, and misunderstandings that can emerge in cross-cultural settings. Here, we address problems arising from such cross-disciplinary, ethnographic work, some of which are similar to those experienced in economic experimental games such as the ultimatum, dictator, or third-party-punishing game (Tracer et al., 2014).

The local conditions to test our planned study on sociality and causality among the Wampar were ideal. The village people are used to having ethnographers who stay for long periods, and ask many different kinds of questions. For instance, the ethnographer had conducted some cognitive tasks on smells during earlier fieldwork (Beer, 2014), which people found entertaining. Many Wampar enjoy doing specific tasks with some interesting material such as samples of smells, colors, or pictures and stories. Some even seem to favor them compared to more general interviews. So, the motivation was good, trust no issue, and nobody approached by the ethnographer refused to answer questions. And still, several different kinds of problems arose. For the subsequent discussion, we tentatively sorted them into three clusters: issues with the *practicability* of task design and execution, issues with data *interpretation*, and issues revolving around *validity.*

### PRACTICABILITY: INDEPENDENCE OF DATA, APPEAL OF TASKS, AND HANDLING OF TASK VERSIONS

It was difficult to get Wampar to sit down and talk *alone*; furthermore, after a few individuals had completed the tasks, it was equally difficulty to find people who had not yet discussed the scenarios extensively with other members of the community. The whole point of routine interaction within the settlement – including with an anthropologist – is for many Wampar precisely the enjoyment of togetherness and casual conversation. Eventually, in the cases documented above, it was possible to create a situation in which only one person was present (at least for some time), listened to the scenarios and answered the questions, although this in itself is already a deviation from naturalistic situations. In several cases children listened or people joined for some time and left again. In case of the questions about the child of an incestuous relationship in Part 2, the laughter of others induced questions and made the interviewer aware of the real case of “Bubu-Dadi.” Here, the reactions of others – which should usually be excluded in experiments – were advantageous because they made clear that many participants had this case in mind when answering the questions. To prevent participants from sharing information and their interpretations after the tasks was impossible: the main value of learning something others have not is exactly in talking about it and sharing the knowledge. So it is likely that some interpretations and ideas about why the ethnographer was interested in helping, deception, and incest would have circulated already and influenced later answers participants gave.

One option for dealing with this problem might be to consider collective sessions as a richer source of relevant discussions and results (and one that might generate more interest and commitment to begin with). However, while this might be a better strategy for grasping local understandings in the pilot phase, it would exacerbate difficulties in data analysis and interpretation within and between cultures were it used for the main study. Given the comparatively small population size, such collective sessions would severely affect sample size – even more so when different versions of the same story had to be discussed with different people or groups of people (between-subjects).

For cognitive psychologists in lab settings, employing tasks like the one used here presents almost no practical issues, even if it takes considerably longer than an hour. When working with the Wampar, however, it became clear that participants could not, or did not want to, concentrate for longer than maximal 30 min. This was particularly obvious in Part 2, where respondents began to confuse persons in the scenario about incest (e.g., the child stolen and taken to town with the child of the incestuous relation), ceased to listen carefully, and even when they understood the questions, did they prefer to talk about different topics (such as the actual case of “Bubu-Dadi,” other people they know or differences between living in town and the village). To be crystal-clear, this is *not* attributed to the Wampars’ ability to concentrate on one task or to stick to a single topic; rather, the observed difficulties must be regarded as arising from the task and/or the way in which it was presented.

This difficulty points toward the more general challenge of how to design tasks in a manner that they appeal to and hold the attention of the people with whom we work. Finding a domain (such as other people’s behavior) and scenarios (such as helping, deceiving, or incest) that are of sufficient interest is a step in this direction, but – as the difficulties faced in our study reveal – only a first step. It may turn out that the abstract examples, and perhaps the set of questions used to structure conversation, did not scaffold the kind of engagement we hoped it would. As ethnographic knowledge is not sufficient, in and of itself, to predict which aspects of a task would be appealing to people, pretesting remains essential – and that implies pretesting in every single cultural context in which the study is to be conducted.

Related to the problem of task duration is the problem of similarities across task variants, especially when, as in the versions of our scenarios, they were planned simply to substitute one pair of kin with another, or aimed at being more or less explicit with some aspects. Participants clearly lost interest in listening to the “same” story several times. They became impatient and the use of otherwise entertaining stories became a chore. Especially *reading* the longer narratives twice turned out to be too unnatural, and people preferred a situation in which the ethnographer ‘read’ or even better ‘told’ them a story and did not only ‘administer a test.’ The atmosphere for the discussion about the series of questions was more relaxed and lively after the narrative when it was read only once and further modifications (as exchanging the mother–son relationship against brother–sister) were explained informally. Bolyanatz (2014, p. 283) describes similar experiences for economic games used in other parts of PNG. This makes controlling specific variables quite difficult, especially when the obvious solution to this problem, namely a between-subject design, is not feasible due to population size.

### INTERPRETATION: CONNECTIONS BETWEEN QUESTIONS, ANSWERS, AND INTERPRETATIONS

More fundamental than the practical problems are concerns with the understanding of the situation and the way the answers match the questions. Attempts to figure out what the researcher has in mind is generally an issue, and perhaps even more so with psychological experiments—where participants expect concealed purposes—than in fieldwork situation once a relationship of trust has been established. But the unfamiliar interaction still requires reconstruction of a common ground for the conversation to be sensible, and this may interfere with the intention of the task (e.g., Stenning, 2012). This problem is amplified by possible differences in conversational conventions, rules of language pragmatics, and/or habits of perspective-taking.

An example that looks simple, *prima facie*, but turns out to be rather complicated, revolves around the pragmatics of responses. For example, in the deception scenario some participants exclaimed “*Em i giaman*,” or “*Em man bilong giaman*.” The first could be translated “He lies,” while the second could suggest that it is his habit to tell lies, or doing so is part of his character, on the basis of the dictionary definition of *giaman.* However, in everyday life, these sentences might be used interchangeably, and only to impugn somebody’s reliability rather than their truthfulness; they might also be used for the pleasure of exaggeration. Other examples could be given.

Several of the answers contain formulations which are sometimes difficult to interpret, including, for example, the simple utterance “I don’t/cannot know” (*mi no klia*, *mi no save*). It is not always clear from the reply if the person wanted to say that s/he cannot answer due to lack of information or comprehension, or that one cannot know in general, or that she/he declines to judge the behavior of others, or has lost interest in the question. Here, our Wampar data connects up with the complex of issues discussed under the heading of opacity of others’ minds and cross-cultural variability (Danziger and Rumsey, 2013 and references therein). This is generally the case in studies of the attribution of motives and causal reasoning about social interactions.

When we asked, for example, what other Wampar would answer if asked the same question, the aim was to access participant’s ideas about shared (and minority) views relevant to behavior. In many cases participants answered, but did not switch perspective; instead they repeated their own opinions and expanded on them. This was not always explicated in their answers, but an impression created in the interviewer, thus highlighting how difficult it can be to assess whether participants actually try to change perspective. When asked about gossip in the incest scenario, for example, many participants continued to think and talk about their own evaluations rather than giving opinions of fellow villagers. Inter-individual differences in the willingness or experience in perspective-taking are an issue as well, especially in cases where participants simply repeated the story (rather than explaining it), shifted perspective from other’s assumed opinion to one’s own, or assumed that the researcher’s fictive story actually was meant as a placeholder for a real event. Participants often referred to their own life-world and personal situation rather than to the scenarios we presented. In a face-to-face community, the micro-politics of relations can rarely be entirely set aside.

Some participants added ideas to the scenarios, which they found important, but which made it difficult to compare them to other answers. For example in the scenario on the incest taboo they speculated on whether the boy earned a lot of money in town. Cole and Scribner (1974), in their study of syllogistic reasoning among non-literate Kpelle of rural Liberia, report that participants were reluctant to stay within problem boundaries: they altered the conditions of the problem to be solved or added personal experiences in order to come to a conclusion. Laypeople in literate societies are also reported to resort to such elaborations when faced with intricate problems, as Henle (1962) reports of American students working to evaluate the adequacy of various syllogistic forms.

Cole and Scribner (1974, p. 166) suggest that these sorts of difficulties have consequences that go beyond the possibility of amelioration through modifications to the tasks presented to participants:

“We cannot draw conclusions about reasoning processes from the *answers* people give to logic problems. We have first to ask: ‘What is their understanding of the task? How do they encode the information presented to them? What transformations does the information undergo, and what factors control these?”’

To give one example from Part 2: when we asked for the characteristics of the baby of the incestuous relationship we aimed at ideas about causal relations between immoral behavior and later events/outcomes. Some participants seemed to assume that the ethnographer meant the specific children of “Bubu-Dadi” (because the ethnographer is interested in interethnic marriages and kin relations) and responded that the child would be okay, meaning mainly “healthy.” Others assumed the question referred to general Christian values, perhaps triggered by the helping/deception scenarios which address topics also discussed at church meetings; according to this frame of interpretation the child is a gift of God, which makes it *per se* “good,” or its characteristics depend on the strength of the belief of the parents^11^. One informant referred in his answer explicitly to *kastom* (tradition, culture) saying the child would be bad. Two other participants responded that they could not and did not know, a definite enough statement, but one that left it unclear whether they thought that the information necessary was omitted from the narrative, or that information about how the moral development of a child will proceed is in principle unobtainable. With a greater number of participants we would face even more of these different interpretive frameworks for interpreting their responses.

This highlights the well-known problem of inter-individual differences, due to the personal histories and/or personalities of participants. These are particulars and this issue raises questions about the relationship between psychological universals and particular cultural contexts.

### VALIDITY: THE “HOME-FIELD DISADVANTAGE” AND SCAFFOLDING

Cross-cultural research, even when anthropologically informed, is an intricate enterprise. In a challenging paper, Medin et al. (2010) discuss issues that contribute to what they label the “home-field disadvantage.” This handicap arises whenever one cultural group (typically the researcher’s own group) is unreflectively taken as the starting point for comparison, and may be manifested as: (1) a tendency to leave one of the cultural groups unmarked, as if it were the standard from which the others differ; (2) a tendency to consider other cultural groups as more homogeneous than the one taken as starting point, and definitely as more homogeneous than they actually are; and (3) an excessive trust in the equivalence of tasks across cultures – both in terms of how these tasks would be understood and responded to by different groups, and in terms of what the obtained data would be able to reveal. If one takes, for instance, a standard psychological task on causal reasoning as the phenomenon of interest, the problem with applying this for cross-cultural research is that this task will have been specifically tailored to bring about a particular effect in the cultural context (typically a WEIRD context), for which it was developed. As a necessary consequence (reasons for which include, among others, regression toward the mean), the same task is unlikely to produce similar results in other cultural contexts (Medin et al., 2010). The antidote recommended by Medin et al. (2010) is a constant effort in marking the unmarked cultural group, collaborating with the group(s) researched, conducting research on the terms of the respective culture, and taking multiple perspectives.

With the approach taken for the current study, this was exactly what was strived for. In order to investigate how people understand and account for the behavior of others conditional upon their relationships, the point of departure was *not* a specific, well-established task from psychological research, but a set of (ethnographically informed) considerations on what the group under study may be willing to talk about. Yet difficulties remain. The most obvious is to figure out how the task should be modified in a way that the Wampar will enjoy, and that would facilitate the type of responses that in turn will help us to answer the questions we have. Some of the experiences reported herein suggest fruitful directions (e.g., replacing individual interviews with collective session, limiting the number of key questions and task versions, finding ways that invite perspective-taking more strongly).

In this context, we wish to explicitly acknowledge a suggestion made by one of our reviewers. As the reviewer stressed, we need to find ways that allow the research to scaffold and enhance the participants’ capacity to report on the processes that govern their considerations. A significant contribution by the ethnographer is thus to illuminate what the participants will be drawn to, what materials are familiar yet multiply interpretable, and what specific ways to representing social life are relevant to the queries at hand. In other words, relationality, historicity, and contextuality need to be accepted as fundamental to any human intention and action (see also Medin et al., 2010; Bloch, 2012) and thus would have to be made an invariable part of any testing milieu. However, as the same conditions should be granted to each participant from every cultural group included in the comparison, the most fundamental challenge will be to create comparable conditions without holding details of the tasks and of the testing context constant.

## CONCLUSION

Laidlaw’s (2007) characterization of the relationship between the anthropology of religion and cognitive science of religion is helpful at this point to clarify some of the problems we have encountered in our study and can partly be transferred to the realm of social interactions more generally. He takes issue with the assumption that cognitive scientists could “explain religion” in terms of basic cognitive processes while what they actually deal with is a limited subset of the features of “religion.” Religions, Laidlaw insists, includes far more complex phenomena grounded in the historically located intentionality of human beings.

In our own study, we tried to investigate how Wampar people draw inferences about social interactions. The prime goal of our study was thus *not* to understand allegedly universal processes in causal inferences about social interactions (helping, deceiving, sexual relations) to be then able to explain causal cognition in general, but to understand the cognitive processes underlying causal inferences in their sociocultural contexts and embedded in social relations. Our study reveals how difficult it can be to get at basic cognitive ‘mechanisms’ or ‘processes’ through fictive scenarios precisely because of the relationality, historicity, and contextuality of people’s intentions and actions.

However, Laidlaw also stresses that – while basic (universal) processes cannot explain complex behavior – their understanding is still an important pre-condition for good general understandings of behavior. In this line, we propose that it is indispensable to try to solve the problems arising when different theoretical and methodical traditions raise meaningful questions and attempt to answer them (for a compelling discussion of both the complications and the inevitability of cross-disciplinary collaboration, see also Bloch, 2012).

Cognitive science needs anthropology in order to substantiate any claims for the universality of cognitive processes (e.g., Astuti and Bloch, 2012; Barrett et al., 2012). Cross-cultural comparisons and the adjustment of research strategies and methods to the social and cultural environments of non-WEIRD populations are essential to achieve this goal. This paper exemplifies this with the description of difficulties encountered in the process of making a cross-cultural experiment relevant and reproducible in different cultural contexts. From an anthropological perspective, long-term fieldwork and naturalistic observation of behaviors with subsequent questioning still appears to be the best choice for getting answers to questions about evaluations of and causal reasoning about social interactions – although these procedures are not in the strict sense of the term ‘reproducible.’ If in-depth knowledge of relations and their history is crucial for understanding, psychologists are well-advised to consider alternatives to the exclusive reliance on quick experiments with a selective sample of people for fast output. As Cole (1978, p. 629, 630) wrote “for the psychologist this position poses the need to develop new techniques in order to study everyday cognitive activities and their relation to the special activities he designs. It also means the loss of certainty about his most trusted tool, the experiment”. Members of the “Laboratory of Comparative Human Cognition” took important steps in this direction, especially Cole and his colleagues in the course of their long collaborative research among the Kpelle.

While the necessities of long-term fieldwork, interdisciplinary processes of developing a methodology and careful cross-cultural testing of methods contradict the political economy of research funding and the academic market, rising to this challenge is the only promising way for real progress in this field.

## AUTHOR CONTRIBUTIONS

The members of the working group on “Causality and Sociality” developed the study concept and the tasks. BB collected and analyzed the data; AB assisted in the analysis. BB and AB wrote the paper.

## Conflict of Interest Statement

The authors declare that the research was conducted in the absence of any commercial or financial relationships that could be construed as a potential conflict of interest.

## Footnotes

1 This fieldwork tied in with previous fieldwork among Wampar in Gabsongkeg by the first author (1997, 1999/2000, 2002, 2003/04, and 2009), and continued a research agenda inspired by the ethnographic work of Hans Fischer, begun in the 1950s. Fischer had conducted fieldwork in Gabmadzung in 1965, and then in Gabsongkeg in 1971/72, 1976, 1988, 1990, 1993, 1997, 1999/2000, 2003/04, and 2009. In 2009/2010, Doris Bacalzo and Tobias Schwörer did research in Dzifasing, and Heide Lienert, Christiana Lütkes, Rita Kramp, and Juliane Neuhaus worked in different Wampar villages (Fischer, 1975, 1996; Beer, 2006).

2 When we write about “the Wampar,” the reader should keep in mind that “Wampar” have not always been a bounded social unit with its own territory. There have been kin groups fighting against each other and moving through the mountains and the Markham Valley. As in other parts of Papua New Guinea “ethnicity is based on continua of cultural difference in a population crisscrossed by flows of people” (Golub, 2014, p. 118).

3 We understand by ‘morality’ collectively sanctioned rules, beliefs and central values that inform the everyday considerations of actors encountering choices and ambivalence in social interactions. These considerations are contextual and relational. Accordingly, we use morality here interchangeably with ethics.

4 That he refused to answer more questions was astonishing, as the boy is otherwise not shy, but very talkative and shares his opinions even on matters which are usually topics for adults. It is, however, in line with some of our other findings and will be discussed below.

5 The ethnographer had the strong impression that this man gave all answers in a way which should correct his negative image and the anticipated critique of his manners, which circulated among Wampar.

6 This was especially a vital lesson to learn for shop owners in the villages who wanted not to give all their goods away to kin, but be able to start a small *bisnis.*

7 A variation of the scenario with a father marrying his daughter, which we had planned as well, was abandoned because it would have been even closer to a real incest case, which people referred to frequently.

8 Many unmarried Wampar women have illegitimate children; they usually live with their parents or a sibling, some have an independent household. That means her needs and the needs of the children (food, school fees) have to be fulfilled by the kin group.

9 This might be due to a specific case which the question led many participants to assume was the ethnographer’s real interest. That actual case might also explain why people reacted less shocked by the narrative on incest than we assumed they would be. During the interviews, it emerged that a Wampar widower married to a woman of another ethnic group just had a fourth child with her, and that, in fact, the woman was his daughter. This widely believed rumor also influenced responses to questions about the offspring of such incestuous relations. Some participants said that “*Bubu-Dadi”* (= Grandfather Daddy) and his daughter had produced healthy children, which they thought of as a proof that incestuous relations *per se* cause no health problems. Generally speaking, participants were easily drawn to volunteer views on the causes and consequences of the case.

10 A 2009 investigation into preferred marriage partners revealed cases of relationships that were seen as incestuous according to Wampar norms. In recent discussions about good and bad marriages, and relations between siblings and cousins, the differentiation between cross and parallel cousins has become less important in norms and practices. As kin-term usage tends to move away from Wampar norms to *Tok Pisin* or English, it seems that certain differentiae (cross-sex/same-sex relative) have become less salient.

11 Even the German nationality of the ethnographer might have had an influence, as the first Lutheran missionaries were German, and although the difference between missionaries and anthropologists is known, there might still be social desirability bias in this answer.
